# Supplementary material for: Geographic variation of mutagenic exposures in kidney cancer genomes
Source: Nature. 2024 May 1;629(8013):910–8. doi: 10.1038/s41586-024-07368-2 (PMC11111402; doi:10.1038/s41586-024-07368-2)
Supplement: Supplementary file 2 — Reporting Summary [file 41586_2024_7368_MOESM2_ESM.pdf]

nature portfolio

Corresponding author(s): Paul Brennan
Last updated by author(s): Feb 16, 2024

Reporting Summary

Nature Portfolio wishes to improve the reproducibility of the work that we publish. This form provides structure for consistency and transparency in reporting. For further information on Nature Portfolio policies, see our Editorial Policies and the Editorial Policy Checklist.

Statistics

For all statistical analyses, confirm that the following items are present in the figure legend, table legend, main text, or Methods section.

n/a	Confirmed	
☐	☒	The exact sample size (*n*) for each experimental group/condition, given as a discrete number and unit of measurement
☐	☒	A statement on whether measurements were taken from distinct samples or whether the same sample was measured repeatedly
☐	☒	The statistical test(s) used AND whether they are one- or two-sided<br>*Only common tests should be described solely by name; describe more complex techniques in the Methods section.*
☐	☒	A description of all covariates tested
☐	☒	A description of any assumptions or corrections, such as tests of normality and adjustment for multiple comparisons
☐	☒	A full description of the statistical parameters including central tendency (e.g. means) or other basic estimates (e.g. regression coefficient) AND variation (e.g. standard deviation) or associated estimates of uncertainty (e.g. confidence intervals)
☐	☒	For null hypothesis testing, the test statistic (e.g. *F*, *t*, *r*) with confidence intervals, effect sizes, degrees of freedom and *P* value noted<br>*Give P values as exact values whenever suitable.*
☐	☒	For Bayesian analysis, information on the choice of priors and Markov chain Monte Carlo settings
☒	☐	For hierarchical and complex designs, identification of the appropriate level for tests and full reporting of outcomes
☐	☒	Estimates of effect sizes (e.g. Cohen's *d*, Pearson's *r*), indicating how they were calculated

Our web collection on statistics for biologists contains articles on many of the points above.

Software and code

Policy information about availability of computer code

Data collection	Whole genome sequencing (150bp paired end) was performed on the Illumina NovaSeq 6000 platform with target coverage of 40X for tumors and 20X for paired blood. REDCap 13.1.27 was used to collect epidemiological data for cases from Barretos, Porto Alegre, Sao Paulo, Leeds, Vilnius, Hat Yai, Belgrade and Bucharest
Data analysis	Algorithms used:<br><br>Variant Calling pipelines (available at https://github.com/cancerit):<br>BWA-Mem v0.7.17-r1188<br>ASCAT v4.3.3 and v4.5.0<br>BATTENBERG v3.5.3<br>cgpCaVEMan v1.11.2, v1.14.1 and v1.15.1<br>cgpPINDEL v2.2.5, v3.3.0 and v3.5.0<br>BRASS v6.1.2, v6.2.0, v6.3.0 and v6.3.4<br>Strelka2 v2.9.10 and Manta 1.6.0<br><br>Other packages:<br>Conpair v0.2 (https://github.com/nygenome/Conpair)<br>SigProfilerMatrixGenerator v1.2.12 (https://github.com/AlexandrovLab/SigProfilerMatrixGenerator)<br>SigProfilerExtractor v1.1.9: (https://github.com/AlexandrovLab/SigProfilerExtractor)<br>MSA v2.0 (https://gitlab.com/s.senkin/MSA)<br>mSigHdp v2.0.1 (https://github.com/steverozen/mSigHdp)

Cancer Genome Interpreter 2022 (https://www.cancergenomeinterpreter.org)
MutationMapper tool v6.0.0 (http://www.cbioportal.org/mutation_mapper)
DPClust R package v2.2.8 (https://github.com/Wedge-lab/dpclust)
dpclust3p v1.0.8 (https://github.com/Wedge-lab/dpclust3p)
SigProfilerAssignment (v0.0.13)
SnpEff 5.0e (https://pcingola.github.io/SnpEff/)
ADMIXTURE v1.3.0 (https://dalexander.github.io/admixture/)
PLINK v1.9 and v2.00a (www.cog-genomics.org/plink/2.0/)
PRSice 2.3.3 (https://choishingwan.github.io/PRSice/)
Profinder 10.0.2.162 (https://www.agilent.com/)
Mass Profiler Professional B.14.9.1 (https://www.agilent.com/)
lme4 1.1-34 (https://github.com/lme4/lme4/)
matrixStats 1.0.0 (https://github.com/HenrikBengtsson/matrixStats)
Matrix 1.6-1.1 (https://matrix.r-forge.r-project.org/)
geojsonio 0.11.3 (https://github.com/ropensci/geojsonio)
ggnewscale 0.4.9 (https://eliocamp.github.io/ggnewscale/)
ggpattern 1.0.1 (https://github.com/trevorld/ggpattern)
ggrepel 0.9.3 (https://github.com/slowkow/ggrepel)
ggsflabel 0.0.1 (https://yutannihilation.github.io/ggsflabel/)
ggspatial 1.1.9 (https://paleolimbot.github.io/ggspatial/)
ggpubr 0.6.0 (https://github.com/kassambara/ggpubr/)
raster 3.6-23 (https://github.com/rspatial/raster)
rgeos 0.6-4 (https://github.com/cran/rgeos/)
sf 1.0-14 (https://github.com/r-spatial/sf)
sp 2.0-0 (https://github.com/edzer/sp/)
tmaptools 3.1-1 (https://github.com/r-tmap/tmaptools)
patchwork 1.1.3 (https://github.com/thomasp85/patchwork)
leaflet 2.2.0 (https://github.com/rstudio/leaflet)
ggplot2 3.4.3 (https://github.com/tidyverse/ggplot2)
cowplot 1.1.1 (https://wilkelab.org/cowplot/)
data.table 1.14.8 (https://github.com/Rdatatable/data.table)
dplyr 1.1.3 (https://github.com/tidyverse/dplyr)
haven 2.5.3 (https://github.com/tidyverse/haven)
Hmisc 5.1-1 (https://hbiostat.org/r/hmisc/)
openxlsx 4.2.5.2 (https://github.com/ycphs/openxlsx)
rgdal 1.6-7 (https://rgdal.r-forge.r-project.org/)
scales 1.2.1 (https://github.com/r-lib/scales)
stringr 1.5.0 (https://github.com/tidyverse/stringr)
tidyr 1.3.0 (https://github.com/tidyverse/tidyr)
tibble 3.2.1 (https://github.com/tidyverse/tibble)
xlsx 0.6.5 (https://github.com/colearendt/xlsx)
rfPermute 2.5.2 (https://github.com/EricArcher/rfPermute)
randomForest 4.7-1.1 (https://www.stat.berkeley.edu/~breiman/RandomForests/)
forcats 1.0.0 (https://github.com/tidyverse/forcats)
jupyter 1.0.0 (https://jupyter.org/)
pandas 1.5.0 (https://pandas.pydata.org/)
numpy 1.23.3 (https://numpy.org/)
scipy 1.9.1 (https://scipy.org/)
statsmodels 0.13.2 (https://www.statsmodels.org/)
firthlogist 0.5.0 (https://github.com/jzluo/firthlogist)
patsy 0.5.2 (https://github.com/pydata/patsy)
matplotlib 3.3.4 (https://matplotlib.org/)
seaborn 0.12.0 (https://seaborn.pydata.org)
plotly 5.10.0 (https://plot.ly)
TMB_plotter (https://github.com/AlexandrovLab/TMB_plotter)

Statistical analysis was performed in R version 4.1 and Python version 3.9.13

For manuscripts utilizing custom algorithms or software that are central to the research but not yet described in published literature, software must be made available to editors and reviewers. We strongly encourage code deposition in a community repository (e.g. GitHub). See the Nature Portfolio guidelines for submitting code & software for further information.

Data

Policy information about availability of data

All manuscripts must include a data availability statement. This statement should provide the following information, where applicable:

- Accession codes, unique identifiers, or web links for publicly available datasets
- A description of any restrictions on data availability
- For clinical datasets or third party data, please ensure that the statement adheres to our policy

Whole genome sequencing data and patient metadata are deposited in the European Genome-phenome Archive (EGA) associated with study EGAS00001003542. Aligned BAM files for all ccRCC cases included in the final analysis were deposited in dataset EGAD00001012102, consensus SNV and indel variant calling files in dataset EGAD00001012222, patient metadata in dataset EGAD00001012223, structural rearrangement variant calling files in dataset EGAD00001013726 and copy number variant calling in dataset EGAD00001013727. Mutational catalogs for the PCAWG dataset can be accessed at https://dcc.icgc.org/releases/PCAWG. Data used for validation of SBS12 in additional cohorts can be retrieved from the original publication (validation cohort 1)32 and EGA dataset EGAD00001009866

(validation cohort 2). The metabolomics data have been uploaded to the MetaboLights repository as study MTBLS9394. The human reference genome used for alignment is available at ftp://ftp.sanger.ac.uk/pub/cancer/support-files/reference/GRCh38_full_analysis_set_plus_decoy_hla.fa. All other data are provided in the accompanying Supplementary Tables.

Research involving human participants, their data, or biological material

Policy information about studies with human participants or human data. See also policy information about sex, gender (identity/presentation), and sexual orientation and race, ethnicity and racism.

Reporting on sex and gender	Sex information was self-reported and collected using epidemiological questionnaires. Overall numbers are provided in the population characteristics section of the Reporting summary. Consent was obtained for sharing individual-level data. Sex-based epidemiological regressions were performed, as described in the Methods section.
Reporting on race, ethnicity, or other socially relevant groupings	To infer the individuals with European genetic background, ADMIXTURE tool and principal component analysis (PCA) were used as described in the Methods section. This variable was not used as a proxy for any other socially constructed variables. Being an unbiased estimate based on genotyping data, no confounding variables were controlled for in the relevant analyses.
Population characteristics	962 cases (380 women and 582 men) diagnosed with ccRCC were included from the following countries: A total of 962 ccRCC cases from 11 countries in four continents were studied, encompassing: Czech Republic (n=259), Russia (n=216), United Kingdom (n=115), Brazil (n=96), Canada (n=73), Serbia (n=69), Romania (n=64), Japan (n=36), Lithuania (n=16), Poland (n=13), and Thailand (n=5). Age of diagnosis ranging from 23 to 87 y.o., mean (SD): 60 (12).
Recruitment	IARC/WHO coordinated cases recruitment through an international network of collaborators in 11 countries. The inclusion criteria for patients were >=18 years of age, confirmed diagnosis of primary renal cell carcinoma (RCC) and no prior treatment. Patients were excluded if they had any condition that could interfere with their ability to provide informed consent or if there were no means of obtaining adequate tissue/ blood samples as per the protocol requirements. The authors are not aware of any potential self-selection bias or other biases present
Ethics oversight	Ethical approvals were obtained from each Local Research Ethics Committee and Federal Ethics Committee as listed below. The study was submitted and approved by the IARC Ethics Committee. Informed consent was obtained from all participants.<br>Barretos Cancer Institute, Barretos, Sao Paulo, Brazil<br>Hospital de Clinicas de Porto Alegre, Porto Alegre, Brazil<br>A.C.Camargo Cancer Center, Sao Paulo, Brazil<br>Ontario Tumor Bank, Canada<br>Charles University in Prague, 2nd faculty of Medicine, Prague, Czech Republic<br>National Cancer Center, Tokyo, Japan<br>National Cancer Institute, Vilnius, Lithuania<br>Nofer Institute of Occupational Medicine, Warsaw, Poland<br>University of Medicine and Pharmacy "Carol Davila", Bucharest, Romania<br>N.N. Blokhin Cancer Research Center, Moscow, Russian Federation<br>International Organization for Cancer Prevention and Research, Belgrade, Serbia<br>Faculty of Medicine Prince of Songkla, Hat Yai, Thailand<br>St James' University Hospital, Leeds, United Kingdom

Note that full information on the approval of the study protocol must also be provided in the manuscript.

Field-specific reporting

Please select the one below that is the best fit for your research. If you are not sure, read the appropriate sections before making your selection.

☒ Life sciences ☐ Behavioural & social sciences ☐ Ecological, evolutionary & environmental sciences

For a reference copy of the document with all sections, see nature.com/documents/nr-reporting-summary-flat.pdf

Life sciences study design

All studies must disclose on these points even when the disclosure is negative.

Sample size	Cases were selected from retrospective and prospective studies from populations which reflect a range of renal cell carcinoma (RCC) incidence rates. Numbers were limited by the number of cases available
Data exclusions	Cases were excluded for any of the following pre-established criteria; 1) Incomplete data on core set variables (age at diagnosis, sex, alcohol and tobacco consumption) 2) Failure to pass pathology review as described in the Methods 3) If matched tumour/normal tissue did not originate from the same individual as determined by Fluidigm SNP genotyping. 4) If sequencing coverage was below 30X for tumour, or 15X for matched normal tissue 5) Evenness of coverage criteria 6) if contamination level was above 3% as determined by Conpair. For evenness of coverage, the median over mean coverage (MoM) score was calculated. Tumors with MoM scores outside the range of values determined by previous studies to be appropriate for whole genome sequencing (0.92 – 1.09) were excluded.
Replication	Signature extraction was replicated two times independently at both Wellcome Sanger Institute and UCSD, with similar results. Signature attribution was replicated two times independently at both Wellcome Sanger Institute and IARC, with similar results. All attempts at replication were successful. No other experiments other than those mentioned here were replicated independently due to limited resources.

Randomization	Randomization is not relevant for this study. Cases did not undergo interventions. All cases were collected based on diagnosis of primary renal cell carcinoma (RCC) and no prior treatment.
Blinding	Blinding is not relevant for this study. Cases were not subject to any interventions.

Reporting for specific materials, systems and methods

We require information from authors about some types of materials, experimental systems and methods used in many studies. Here, indicate whether each material, system or method listed is relevant to your study. If you are not sure if a list item applies to your research, read the appropriate section before selecting a response.

Materials & experimental systems

n/a	Involved in the study
☒	☐ Antibodies
☒	☐ Eukaryotic cell lines
☒	☐ Palaeontology and archaeology
☒	☐ Animals and other organisms
☒	☐ Clinical data
☒	☐ Dual use research of concern
☒	☐ Plants

Methods

n/a	Involved in the study
☒	☐ ChIP-seq
☒	☐ Flow cytometry
☒	☐ MRI-based neuroimaging
